# Exploration of Mechanochemical Activation in Solid-State Fluoro-Grignard Reactions

**DOI:** 10.3390/molecules25030570

**Published:** 2020-01-28

**Authors:** Isaiah R. Speight, Timothy P. Hanusa

**Affiliations:** Department of Chemistry, Vanderbilt University, Nashville, TN 37235, USA; isaiah.r.speight@vanderbilt.edu

**Keywords:** mechanochemistry, organic chemistry, organofluorine, Grignard reaction, ball milling

## Abstract

Owing to the strength of the C–F bond, the ‘direct’ preparation of Grignard reagents, i.e., the interaction of elemental magnesium with an organic halide, typically in an ethereal solvent, fails for bulk magnesium and organofluorine compounds. Previously described mechanochemical methods for preparing Grignard reagents have involved ball milling powdered magnesium with organochlorines or bromines. Activation of the C–F bond through a similar route is also possible, however. For example, milling 1- and 2-fluoronaphthalene with an excess of magnesium metal for 2 h, followed by treatment with FeCl_3_ and additional milling, produces the corresponding binaphthalenes, albeit in low yields (ca. 20%). The yields are independent of the particular isomer involved and are also comparable to the yields from corresponding the bromonaphthalenes. These results may reflect similar charges that reside on the α-carbon in the naphthalenes, as indicated by density functional theory calculations.

## 1. Introduction

For well over a century, the indispensable Grignard reagents have played critical roles in the synthetic chemist’s toolbox, and the range of reactions they facilitate is enormous [1,2,3]. Various methods have been used to promote the so-called ‘direct’ preparation of Grignard reagents, i.e., the interaction of elemental magnesium with an organic halide, usually in an ethereal solvent [4]. The application of ultrasound [5,6] or microwave irradiation [7], and the formation of finely divided metal powders, have all been used to boost the reactivity of elemental magnesium. Exploration of the latter approach was pioneered by Rieke, who prepared highly reactive metals by reducing metal salts with alkali metals or organoalkalis in ethereal or hydrocarbon media [8,9,10,11,12,13,14]. Peripherally related to this technique, in that exceptionally small metal particles are involved, is mechanochemical activation of reactions, typically achieved through grinding or ball milling. The process provides energy input without requiring the use of solvents or the application of elevated temperatures [15,16,17,18,19,20]. Although the literature on the subject is not large, mechanochemical methods have been employed in Grignard chemistry with various degrees of success.

**Scheme 1:**
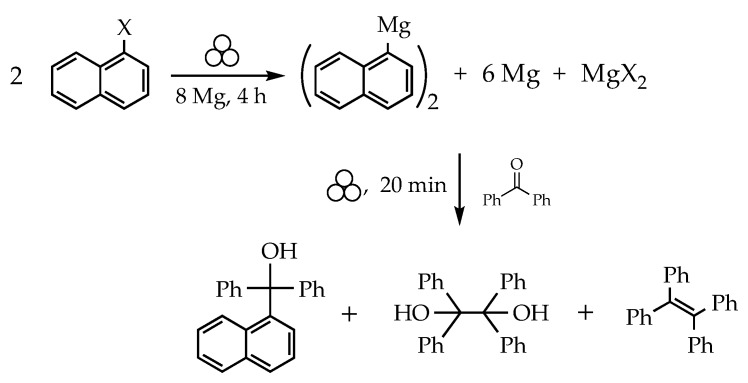
Products of the milling of magnesium (Mg) with halogenonaphthalenes (X = Cl, Br) [26]. The tertiary alcohol is the expected product from the Grignard reagent; the diol and alkene are consequences of McMurry coupling from the large amount of finely divided Mg in the reaction. This scheme follows the proposal of Kaupp [27], who suggested that the organomagnesium intermediate operative in this system is likely di(napthyl)magnesium, rather than a napthylmagnesium halide. The symbol for mechanochemical reaction conditions is adopted from ref. [16].

Mechanochemical approaches have sometimes involved the use of preformed Grignard reagents [21], and Mack determined that as long as the grinding vessel was sealed, the milling of Grignard reagents and substrates need not be performed in rigorously anhydrous or anerobic environments [22]. The actual generation and subsequent use of Grignard reagents under mechanochemical conditions has been studied in the context of dehalogenation reactions. Complete dechlorination of 1,3,5-trichlorobenzene, for example, is achieved when it is milled with magnesium and *n*-butyl amine. The process involves the stepwise formation of the corresponding Grignard reagents, for which the amine serves as a hydrogen donor, ultimately generating benzene [23]; other organochlorines have been similarly investigated [24,25]. In the search for more general synthetic applications, Harrowfield reported the solvent-free reaction of magnesium with halogenonaphthalenes in a ball mill [26]. The use of at least a four-fold excess of magnesium was required to produce a manipulable solid, instead of an intractable paste. Not surprisingly, the presence of the highly reactive excess magnesium powder complicated further reactions. When quenched with aromatic ketones, for example, McMurry coupling occurred in addition to the alcohol formation expected from reaction with the organomagnesium species (Scheme 1).

Despite the somewhat uncertain prospects for the clean mechanochemical generation of an RMgX species, we were interested in investigating Grignard systems that do not usually work well—if at all—in solution, specifically fluoro-Grignards (‘RMgF’). The C–F bond is roughly 38 kcal mol^−1^ stronger than the next strongest carbon-halogen bond (C–Cl) [28], and the attempted reaction of elemental magnesium with an organofluorine in solution is usually unsuccessful [29]. Exploration of this issue dates back a century, beginning with the work of Swarts in 1921 [30], and an array of methods has been used in efforts to provide a more reactive magnesium source [31]; some of the early strategies have been reviewed [32].

It should be noted that the preparation of fluoro-Grignard reagents has been described via indirect routes from organomagnesium compounds, including other Grignard reagents [33]. For example, the reaction of EtMgBr with perfluoraryl compounds in the presence of certain transition metal halide catalysts (e.g., CoCl_2_, NiCl_2_, and CuI) was used to generate the corresponding ArMgF species that then underwent the expected Grignard reactions [34]. Similarly, the reaction of MgR_2_ (R = Me, Et, Bu, and Ph) with fluorinating agents such as BF_3_∙OEt_2_, Bu_3_SnF, and SiF_4_ produced the associated RMgF species, although not always in high purity [32]. Crabtree reported the use of the thermally sensitive magnesium anthracene (MgC_14_H_10_) to activate perfluorinated alkyl or aryl compounds, followed by reaction with CO_2_ to produce carboxylic acids, albeit in low to moderate yields (6–34%). Reaction of the same organofluorines with elemental magnesium yielded no product, nor did the reaction of perfluoronapthalene with (MgC_14_H_10_) [35].

Despite these reports, the lure of a direct route to fluoro-Grignards remains, as it would avoid the need for prior synthesis of an organomagnesium reagent that might not be readily accessible, and hence the potential range of R groups could be larger. Rieke’s first work with finely divided magnesium (Mg) focused on organochlorines and -bromines, but fluorobenzene was examined as well [8]. The refluxing of fluorobenzene with Mg in diglyme for 1 h, followed with treatment with CO_2_, produced benzoic acid in very low yield (ca. 5%) [36,37]. Evidence for the formation of fluoro-Grignards by these bulk synthetic methods is indirect, e.g., the formation of the expected reaction products (e.g., after hydrolysis or treatment with CO_2_), which is consistent the formation of RMgF species as intermediates. For the sake of completeness, it could be mentioned that magnesium vapor has been used to prepare fluoro-Grignards at low temperatures. For example, when excited state (^3^P) magnesium atoms produced from laser ablation experiments were allowed to react with methyl halides diluted in an argon matrix, Grignard molecules CH_3_MgX, including the fluoride species, CH_3_MgF, were identified [38]. Similarly, cluster Grignard reagents (C_6_H_5_Mg_n_X), including the combination *n* = 4, X = F, have been produced by metal vapor synthesis [39,40]. Finally, although it does not involve the reaction of organometallic compounds, it might be noted that the reaction of (BDI)Mg–Mg(BDI) (BDI = κ^2^-{2,6-^i^Pr_2_C_6_H_3_NCMe}_2_CH) [41,42] with a series of perfluorinated and polyfluorinated arenes generates (BDI)MgF by a process deemed ‘equivalent’ to Grignard formation in solution [43].

We report here the use of the mechanochemical activation of Mg with fluorinated naphthalenes, in which it is clear that C–F bond activation has occurred, although there are still practical issues that must be overcome prior to more general development of the method. There have been cautions raised about the potentially explosive nature of fluorinated Grignards [29], but no such difficulties were encountered with the monofluoro organics used here.

## 2. Results and Discussion

### 2.1. Exploration of Mechanochemically Generated Grignard Reagents

Owing to the paucity of literature on mechanochemically driven Grignard chemistry, and to the potential sensitivity of reaction outcomes to the specific equipment and conditions employed, we selected several homocoupling reactions as calibration points for reactivity. In these cases, there are no other potential organic products other than ones generated from the Grignard reagents themselves. For this initial study, aromatic substrates were chosen as more likely to provide greater reactivity than the saturated equivalents [35]. A coupling reagent whose efficacy has been established in solution chemistry (FeCl_3_) was chosen [44,45], and bromobenzene was used as the substrate. As noted above, Harrowfield found that a considerable excess (at least 4× stoichiometric levels) of magnesium metal was required when milling it in Grignard reactions [26]. The use of smaller amounts led to pasty mixtures that could not be easily manipulated. We found a similar situation to be true, but perhaps because of our use of finer Mg powder (325 mesh) than in Harrowfield’s experiments (50 mesh), eight equiv. of Mg were required to produce a friable reaction product. Milling experiments were carried out using 100 mg of PhBr, eight equiv. of Mg, and one equiv. of FeCl_3_, performed under the conditions stated in Section 3.1. The coupled product biphenyl was generated in reasonably good yield (Equation (1)). It should be noted that biphenyl is a common contaminant in solution reactions involving PhMgBr [46], but it was not observed under these conditions in the absence of FeCl_3_.

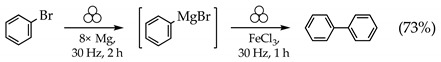
(1)

For the initial screening of an organofluorine, 2-fluoronapthalene was selected. Milling it with eight equiv. of Mg for 2 h left a black powder that, could be extracted with THF. The extract was then filtered, and the filtrate treated with water, resulting in the production of naphthalene in 79% isolated yield (Equation (2)); this is similar to the yield of naphthalene reported from milling magnesium with 1-bromo- and 1-chloronapthalene (95%) [26], although in the latter case the yields were estimated from GC-MS data.

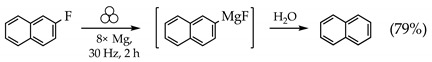
(2)

In order to examine more completely the reactivity differences between two sets of halogenated compounds, 1- and 2- bromo- and fluoronaphthalenes were treated with magnesium under the same conditions as used for bromobenzene (Table 1). Regardless of the halide used, the yield of the isolated product binapthalene was almost identical—roughly 20%.

Curiously, and in line with other reports about of the low reactivity of RMgF species towards CO_2_ [35,36], the presumptive fluoro-Grignards described here were relatively unreactive toward carbonyl addition. Generation of the Grignard reagent was followed by either mechanochemical quenching or treatment of a solution of an electrophile (i.e., benzaldehyde, benzophenone) with the ground powder in order to minimize pinacol and McMurray-type coupling from the excess magnesium powder, yet no product was isolated. Addition of Et_2_O as a liquid-assisted grinding (LAG) agent [47,48,49] also gave no product formation. The addition of lithium chloride to make a Turbo-Grignard reagent (RMgX-LiCl) and the addition of Lewis acids, such as BF_3_∙OEt_2_, which doubles as an LAG agent, were not successful (see Supplementary Materials). There may be as yet not understood matrix effects that are interfering with subsequent reactivity.

### 2.2. Charge Analysis of Grignard Reagents

**Figure 1:**
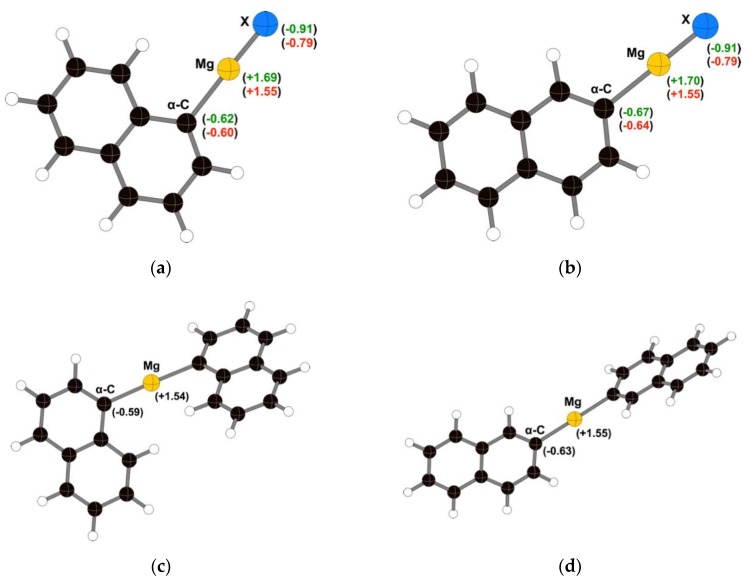
Natural Population Analysis (NPA) charges on the halogen, Mg, and α-C in (**a**) naphthalen-1-ylmagnesium bromide (fluoride); (**b**) naphthalen-2-ylmagnesium bromide (fluoride); (**c**) bis(1-napthyl)magnesium; and (**d**) bis(2-napthyl)magnesium. In (**a**) and (**b**), the top values (in green) correspond to the fluoro species, and the bottom values (in red) to the bromo compounds. The (napthyl)_2_Mg complexes were optimized under C_2_ symmetry, so the charges on both α-carbons in each molecule are the same.

Geometry optimization of the (napthyl)MgX and (napthyl)_2_Mg compounds was conducted with the dispersion-corrected B3PW91-D3BJ functional and the def2TZVP basis set on all atoms (see Section 3.3 for details). Charge estimation was conducted under the Natural Population Analysis (NPA) protocol (Figure 1). Interestingly, the Mg atom (or MgX unit) seems to buffer the charge on the α-carbon in each species. In both halonapthalenes, the difference in the carbon change between the fluoro and bromo variants is no more than 0.03 units. Although there is a slightly more negative charge on the α-carbons in 2-halonapthalene than in the 1-halo isomer, it does not exceed 0.05 units. The pattern in the (napthyl)_2_Mg species is similar, in that the charge on the α-carbons in the two complexes differs by no more than 0.04 units.

The α-carbon charges for all the molecules fall in the range of −0.63 ± 0.04. Accordingly, it should not be surprising that little difference is observed between the reactivity of the fluoro- and bromo- species if, once the bond to Mg is broken, the nucleophilic carbon is effectively unbiased toward the identity of the halogen that was present prior to activation. It also suggests that it may be difficult to distinguish between a (napthyl)MgX or a (napthyl)_2_Mg species in the coupling reactions.

## 3. Materials and Methods

Unless otherwise noted, all manipulations were performed with the exclusion of air and moisture using Schlenk or glovebox techniques. Bromobenzene (purity ≥98%), 1-bromonaphthalene (purity ≥98%), 1-fluoronaphthalene (purity ≥98%), 2-bromonaphthalene (purity ≥98%), and 4-fluorotoluene (purity 97%) were purchased from Oakwood Products, Inc. (Estill, SC, USA), and 2-fluoronaphthalene (purity ≥98%) was purchased from Fisher Scientific (Pittsburgh, PA, USA). Bromobenzene, 1-bromonaphthalene, 1-fluoronaphthalene, and 4-fluorotoluene were all degassed and stored under a nitrogen atmosphere without further manipulation. Magnesium powder (purity 99%, ~325 mesh) was purchased from Strem Chemicals (Newburyport, MA, USA) and stored under a nitrogen atmosphere without further manipulation. Iron(III) chloride was purchased from Sigma-Aldrich (St. Louis, MO, USA), dried under vacuum over an oil bath, and stored under nitrogen. Toluene was degassed with argon and dried over activated alumina, then stored over 4A molecular sieves in a nitrogen atmosphere glovebox. Anhydrous tetrahydrofuran (THF) was stored over 4A molecular sieves in a nitrogen atmosphere glovebox. CDCl_3_ was obtained from Cambridge Isotopes.

### 3.1. General Procedure for Homocoupling Reactions

In a typical procedure, a 15 mL stainless steel Form-Tech milling jar was loaded with two 8 mm stainless steel (440 grade) ball bearings (3.3 g each). In a nitrogen atmosphere glovebox, the aryl halide (100 mg, one equiv.) and the magnesium powder (eight equiv.) were added to the milling jar. The jar was sealed tightly, and electrical tape was used to protect the atmosphere inside the milling jar. The jar was removed from the glovebox, placed in a Retsch MM 400 mixer mill, and milled for the specified time and frequency (2 h, 30 Hz). The jar was returned to the glovebox in a c-clamp, and FeCl_3_ (one equiv.) was added to the ground reaction mixture. The jar was resealed, removed from the glovebox, and remilled (1 h, 30 Hz). Upon being returned to the glovebox, the ground mixture was extracted with toluene (ca. 60 mL) and filtered through a medium porosity ground glass frit. After standard workup to isolate the products, the crude materials were purified using column chromatography with hexanes as the eluent. The dried isolated products were identified by their characteristic ^1^H-NMR spectra on a Bruker AV-400 spectrometer (Billerica, MA, USA) at 400 MHz [50,51,52].

### 3.2. General Procedure for Quenching Reactions

The procedure above was followed, but following the initial milling reaction, the material was extracted with THF (ca. 40 mL), and the filtrate treated with 15 mL of deionized water. Workup was as above, but no column purification was used. Products were identified by their characteristic ^1^H-NMR spectra on a Bruker AV-400 spectrometer at 400 MHz [53].

### 3.3. Procedures for Computational Analysis

All calculations were performed with the Gaussian 16W suite of programs [54]. The B3PW91 functional, which incorporates Becke’s three-parameter exchange functional with the 1991 gradient-corrected correlation functional of Perdew and Wang, was used [55]. To add dispersion correction, Grimme’s D3 correction [56] with additional Becke-Johnson damping was used [57] (Gaussian keyword: empiricaldispersion = GD3BJ). The def2TZVP basis set was used on all atoms [58]. The (napthyl)MgX molecules were optimized under C_s_ symmetry, and the (napthyl)_2_Mg complexes were run under C_2_ symmetry. Atomic charges were estimated with the Natural Population Analysis protocol (v 3.1) [59,60].

## 4. Conclusions

Mechanochemically induced Grignard reagent formation by the direct ball milling of magnesium metal and an organohalogen (X = Cl, Br) has previously been demonstrated to provide products and yields comparable to those obtained in solution reactions [26]. Extension of this technique to fluoronapthalenes has now been shown to induce activation of the C–F bond as well, although yields of, e.g., homocoupled naphthalenes under the conditions used here are low (ca. 20%). Density functional theory calculations indicate that charges on the α-carbon in the naphthalenes are similar in all cases. Thus, although it is clear that activation of the C–F bond of the fluoronapthalenes has occurred, whether a (napthyl)MgF or a (napthyl)_2_Mg species is involved in the coupling reactions is uncertain. Mechanochemical promotion of Grignard reagents offers many other variables in the search for improved conditions (e.g., magnesium source, composition of the grinding balls, type of milling apparatus, temperature of the milling, etc.), and thus it is likely that further optimization of reaction conditions is possible.

## Figures and Tables

**Table 1:** Comparison of halide and isomer reactivity in homocoupling reactions with mechanochemically generated Grignard reagents ^1^.

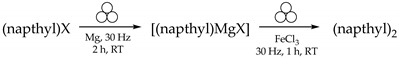	
**Aryl Halide**	**Isolated Yield**
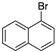	22%
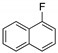	20%
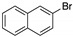	21%
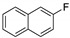	22%

^1^ Conditions: halonapthalene (one equiv.), Mg powder (eight equiv.), FeCl_3_ (one equiv.), two 8mm stainless steel ball bearings (3.3 g ball^−1^), N_2_ atmosphere, and isolated yields after column chromatography.

## Supplementary Materials

The following are available online at https://www.mdpi.com/1420-3049/25/3/570/s1: General procedures and specific details for carbonyl addition reactions, and coordinates of geometry-optimized structures.
