# Supplementary material for: Exploration of Mechanochemical Activation in Solid-State Fluoro-Grignard Reactions
Source: Molecules. 2020 Jan 28;25(3):570. doi: 10.3390/molecules25030570 (PMC7037680; doi:10.3390/molecules25030570)
Supplement: Supplementary file 1 [file molecules-25-00570-s001.pdf]

SUPPORTING INFORMATION

FOR

Exploration of Mechanochemical Activation in Solid State Fluoro-Grignard Reactions

Isaiah R. Speight [a] and Timothy P. Hanusa[a],*

[a]Department of Chemistry, Vanderbilt University, VU Station B #351822,
Nashville, Tennessee 37235 USA

General Procedure for Carbonyl Addition Reactions S2

Table S1: Carbonyl addition attempts with mechanochemically generated Grignard reagents . S3

Coordinates of geometry-optimized structures: (1-naph)MgX, (2-naph)MgX, $(1\text{-naph})_2Mg$, and $(2\text{-naph})_2Mg$ S4

General Procedure for Carbonyl Addition Reactions

In a typical procedure, a 15 mL stainless steel Form-Tech milling jar was loaded with two 8 mm stainless steel (440 grade) ball bearings (3.3 g each). Magnesium powder (325 mesh, 8 equiv.) and the aryl halide (100 mg, 1 equiv.) were added to the milling jar. The jar was sealed tightly, and after grinding in a Retsch MM 400 mixer mill (2 h, 30 Hz), was returned to the glovebox, and the carbonyl electrophile (see Table S1 for specifics) was added to the ground reaction mixture. The jar was resealed and remilled (1 h, 30 Hz). The ground mixture was extracted with ~40 mL of dry THF and filtered through a medium porosity ground glass fritted funnel. The filtrate was treated with 15 mL of saturated ammonium chloride along with 15 mL of toluene. The mixture was transferred into a separatory funnel and the layers were separated. The organic layer was then washed with a 15 mL portion of deionized water. The aqueous layers were extracted once with 15 mL of toluene, the organics combined and washed with a 15 mL portion of brine. The organics were then collected, dried over magnesium sulfate, and filtered into a 100 mL round bottomed flask. The solvent was removed by rotary evaporator and the crude material was purified by column chromatography (hexanes:ethyl acetate) to reveal unreacted electrophile and other unidentified species.

Table S1. Carbonyl addition attempts with mechanochemically generated Grignard reagents.

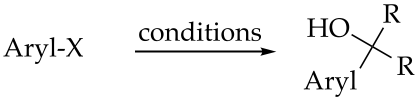

Aryl Fluoride	Electrophile	Deviations from General Procedure	Reaction Time (1st step / 2nd step)
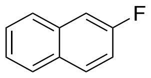	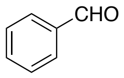	None	2 h / 15 min
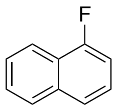	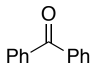	Three 8 mm stainless steel ball bearings (3.3 g ball^{-1}) were used. Second step milled at 15 Hz.	2 h / 15 min
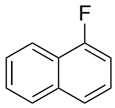	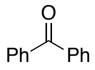	Three 8 mm stainless steel ball bearings (3.3 g ball^{-1}) were used. First and second step milled at 15 Hz.	2 h / 15 min
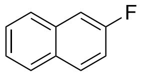	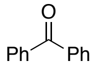	Second step stirred in THF with electrophile.	2 h / 1 h
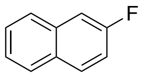	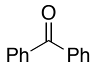	Second step stirred in diethyl ether w/ 0.7 equiv. electrophile.	2 h / 4 h
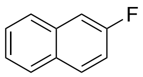	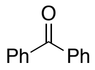	First step milled w/ 2.25 equiv. LiCl. Second step stirred in diethyl ether with electrophile.	2 h / 1 h
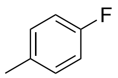	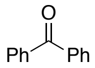	First step milled w/ 0.5 equiv. $BF_3 \cdot OEt_2$. Second step stirred in THF w/ 0.5 equiv. electrophile.	1 h / 1 h
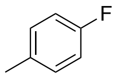	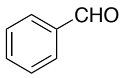	First step milled w/ 0.5 equiv. $BF_3 \cdot OEt_2$. Second step stirred in THF w/ 0.5 equiv. electrophile.	1 h / 1 h
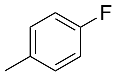	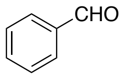	First step milled w/ 0.5 equiv. $BF_3 \cdot OEt_2$. Second step stirred in THF w/ 0.5 equiv. electrophile.	2 h / 1 h
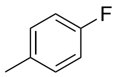	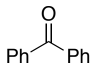	First step milled w/ 0.5 equiv. $AlCl_3$. Second step stirred in THF w/ 0.5 equiv. electrophile.	1 h / 48 h
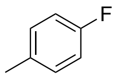	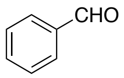	First step milled w/ 0.5 equiv. $AlCl_3$. Second step stirred in THF w/ 0.5 equiv. electrophile.	1 h / 48 h

Coordinates of geometry-optimized structures (B3PW91-D3BJ/def-TZVP)

**(1-Napth)MgF**	E = -685.2725495 au		
C	-1.694462	2.132906	0.000000
C	-1.349022	0.808442	0.000000
C	0.000000	0.383273	0.000000
C	1.007829	1.394115	0.000000
C	0.627998	2.754996	0.000000
C	-0.690998	3.120149	0.000000
H	-2.737978	2.424219	0.000000
H	-2.140610	0.059085	0.000000
C	0.355035	-0.999560	0.000000
C	2.364658	1.002361	0.000000
H	1.405979	3.511030	0.000000
H	-0.967609	4.167702	0.000000
C	2.704353	-0.321758	0.000000
C	1.699938	-1.312292	0.000000
H	3.130637	1.770362	0.000000
H	3.748127	-0.615519	0.000000
H	2.025210	-2.349610	0.000000
Mg	-1.121802	-2.447352	0.000000
F	-2.350455	-3.708316	0.000000

**(1-Napth)MgBr**	E = -3159.6043071 au		
C	3.044587	1.143825	0.000000
C	1.735420	0.744593	0.000000
C	1.366701	-0.620956	0.000000
C	2.417361	-1.586830	0.000000
C	3.761529	-1.151502	0.000000
C	4.072002	0.181413	0.000000
H	3.293132	2.198319	0.000000
H	0.954148	1.505060	0.000000
C	0.000000	-1.033111	0.000000
C	2.080613	-2.958515	0.000000
H	4.548895	-1.897743	0.000000
H	5.107255	0.500960	0.000000
C	0.771405	-3.352012	0.000000
C	-0.259426	-2.388927	0.000000
H	2.879102	-3.692626	0.000000
H	0.520268	-4.406836	0.000000
H	-1.282944	-2.755056	0.000000
Mg	-1.494384	0.393345	0.000000
Br	-3.200811	1.998855	0.000000

**(2-Napth)MgF**		E = -685.270615	
C	-3.353126	-1.674406	0.000000
C	-2.090985	-2.204436	0.000000
C	-0.955493	-1.364634	0.000000
C	-1.140343	0.045475	0.000000
C	-2.455887	0.561051	0.000000
C	-3.537757	-0.277341	0.000000
H	0.509303	-2.944942	0.000000
H	-4.217099	-2.328488	0.000000
H	-1.945636	-3.279256	0.000000
C	0.363090	-1.869457	0.000000
C	0.000000	0.883432	0.000000
H	-2.594408	1.637029	0.000000
H	-4.541909	0.130065	0.000000
C	1.292255	0.393492	0.000000
C	1.439210	-1.022976	0.000000
H	-0.192373	1.954990	0.000000
H	2.432266	-1.464277	0.000000
Mg	2.900952	1.687924	0.000000
F	4.263626	2.802066	0.000000

**(2-Napth)MgBr**		E = -3159.6022192	
C	-5.076790	0.144234	0.000000
C	-4.191758	1.188671	0.000000
C	-2.799020	0.954556	0.000000
C	-2.325112	-0.386539	0.000000
C	-3.264228	-1.442194	0.000000
C	-4.608562	-1.184807	0.000000
H	-2.209115	3.027239	0.000000
H	-6.143276	0.336051	0.000000
H	-4.549367	2.212559	0.000000
C	-1.852388	2.002239	0.000000
C	-0.928990	-0.617043	0.000000
H	-2.900498	-2.464192	0.000000
H	-5.319044	-2.002988	0.000000
C	0.000000	0.406033	0.000000
C	-0.509440	1.735509	0.000000
H	-0.614077	-1.659124	0.000000
H	0.176287	2.578452	0.000000
Mg	2.018717	-0.020021	0.000000
Br	4.304920	-0.531192	0.000000

**$(1\text{-Napth})_2Mg$**		E = -970.6598966	
C	0.661013	4.256252	-2.364660
C	0.102360	4.902032	-1.294841
C	-0.239851	4.200607	-0.116925
C	0.000000	2.795409	-0.040294
C	0.581296	2.171988	-1.170082
C	0.904787	2.871167	-2.301716
H	-0.998792	5.920723	0.939262
H	0.918150	4.807242	-3.261542
H	-0.086644	5.969575	-1.337049
C	-0.816533	4.852809	0.995170
C	-0.337804	2.054776	1.134116
H	0.782181	1.101325	-1.140495
H	1.349130	2.362057	-3.148699
C	-0.899341	2.758002	2.181851
C	-1.138834	4.147153	2.121054
H	-1.178135	2.246319	3.100186
H	-1.581656	4.654056	2.971509
Mg	0.000000	0.000000	1.169132
C	0.337804	-2.054776	1.134116
C	0.000000	-2.795409	-0.040294
C	0.899341	-2.758002	2.181851
C	-0.581296	-2.171988	-1.170082
C	0.239851	-4.200607	-0.116925
C	1.138834	-4.147153	2.121054
H	1.178135	-2.246319	3.100186
C	-0.904787	-2.871167	-2.301716
H	-0.782181	-1.101325	-1.140495
C	-0.102360	-4.902032	-1.294841
C	0.816533	-4.852809	0.995170
H	1.581656	-4.654056	2.971509
C	-0.661013	-4.256252	-2.364660
H	-1.349130	-2.362057	-3.148699
H	0.086644	-5.969575	-1.337049
H	0.998792	-5.920723	0.939262
H	-0.918150	-4.807242	-3.261542

**(2-Napth)$_2$Mg**		E = -970.6556937	
C	0.000000	7.038719	-0.548307
C	0.767552	6.299791	0.311463
C	0.624573	4.896562	0.383460
C	-0.331727	4.256784	-0.452660
C	-1.108282	5.047731	-1.328965
C	-0.947946	6.406291	-1.377315
H	2.126697	4.580162	1.895682
H	0.118710	8.114894	-0.594785
H	1.498082	6.783652	0.950826
C	1.395376	4.096789	1.255375
C	-0.471159	2.850296	-0.377251
H	-1.837075	4.557432	-1.965833
H	-1.549741	7.001732	-2.053892
C	0.279894	2.060720	0.473622
C	1.226427	2.738444	1.294875
H	-1.216063	2.407514	-1.037398
H	1.846151	2.170595	1.984385
Mg	0.000000	0.000000	0.502538
C	-0.279894	-2.060720	0.473622
C	0.471159	-2.850296	-0.377251
C	0.331727	-4.256784	-0.452660
C	-0.624573	-4.896562	0.383460
C	-1.395376	-4.096789	1.255375
C	-1.226427	-2.738444	1.294875
H	1.837075	-4.557432	-1.965833
H	1.216063	-2.407514	-1.037398
C	1.108282	-5.047731	-1.328965
C	-0.767552	-6.299791	0.311463
H	-2.126697	-4.580162	1.895682
H	-1.846151	-2.170595	1.984385
C	0.000000	-7.038719	-0.548307
C	0.947946	-6.406291	-1.377315
H	-1.498082	-6.783652	0.950826
H	-0.118710	-8.114894	-0.594785
H	1.549741	-7.001732	-2.053892
